# Search for putative gene regulatory motifs in CAHS3, linked to anhydrobiosis in a tardigrade *Ramazzottius varieornatus*, in vivo and in silico

**DOI:** 10.1111/gtc.13168

**Published:** 2024-09-30

**Authors:** Sora Ishikawa, Sae Tanaka, Kazuharu Arakawa

**Affiliations:** ^1^ Institute for Advanced Biosciences Keio University Tsuruoka Japan; ^2^ Systems Biology Program, Graduate School of Media and Governance Keio University Fujisawa Japan; ^3^ Exploratory Research Center on Life and Living Systems (ExCELLS) National Institutes of Natural Sciences Okazaki Japan; ^4^ Faculty of Environment and Information Studies Keio University Fujisawa Japan

**Keywords:** anhydrobiosis, gene expression, regulatory sequence, tardigrades

## Abstract

Tardigrades possess the ability to enter an almost completely dehydrated state, anhydrobiosis. The CAHS (cytosolic abundant heat‐soluble) protein family has been identified as one of the anhydrobiosis‐related proteins. In particular, CAHS3 protein from an anhydrobiotic tardigrade, *Ramazzottius varieornatus*, shows heat‐solubility and reversible condensation and is one of the most highly expressed among the CAHS paralogs. A recently developed tardigrade‐specific vector showed tissue‐specific expression of RvCAHS3 most pronounced in the epidermis in vivo, contrary to the idea that anhydrobiotic genes are uniformly expressed in all tardigrade cells. In this study, we investigated the regulation of RvCAHS3 gene expression through in vivo expression experiments using tardigrade vectors with a series of truncated upstream regions coupled with in silico analysis to identify the anhydrobiosis‐related genes that are expressed under the same regulatory system as RvCAHS3. As a result, the 300–350 bp region upstream of RvCAHS3 is critical for regulating gene expression in tardigrade vector experiments, and three motifs conserved between two species of anhydrobiotic tardigrades were identified within a 500 bp region directly upstream of RvCAHS3 start codon. These motifs, which have also been identified upstream of other CAHS genes, could be associated with the regulatory system of anhydrobiosis‐related genes in tardigrades.

## Graphical abstract

Tardigrades can survive in an almost completely dehydrated state, known as anhydrobiosis, aided by proteins like CAHS3. Research using a newly developed tardigrade‐specific vector revealed that CAHS3 expression is most pronounced in the epidermis, suggesting tissue‐specific regulation rather than uniform expression. The study identified critical upstream regions and conserved motifs that likely regulate CAHS3 and other anhydrobiosis‐related genes in tardigrades.

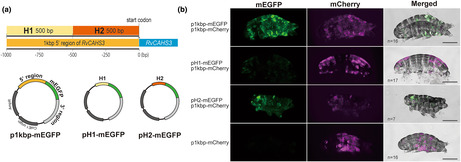

## 1 INTRODUCTION

Water is an essential component of life; however, some microscopic invertebrates, including tardigrades, can tolerate complete desiccation by entering a reversible anhydrobiotic state (Crowe et al., 1992). Tardigrades exhibit remarkable variation in desiccation tolerance; in Parachela, one order of tardigrades in the class Eutardigrada, *Ramazzottius varieornatus* is able to enter an anhydrobiotic state within 30 min and thus tolerate rapid desiccation (Horikawa et al., 2008), whereas *Hypsibius exemplaris* can only tolerate moderate speed desiccation and requires 24 h of pretreatment at high relative humidity to successfully enter the anhydrobiotic state (Kondo et al., 2015). In the anhydrobiotic state, tardigrades are extremely tolerant to various stress conditions, such as temperatures from near absolute zero to 100°C (Becquerel, 1950; Hengherr et al., 2009), high pressures up to 7.5 GPa (Ono et al., 2008), high‐dose irradiation (Altiero et al., 2011; Beltrán‐Pardo et al., 2013, 2015; Charlotta Nilsson et al., 2010; Horikawa et al., 2008; Ingemar Jönsson et al., 2005; Jönsson & Wojcik, 2017; May et al., 1964), and even exposure to space vacuum (Jönsson et al., 2008; Persson et al., 2011; Rebecchi et al., 2009).

CAHS (Cytosolic abundant heat‐soluble) family of proteins were the first set of tardigrade‐specific genes linked to the molecular mechanisms of anhydrobiosis (Arakawa, 2022; Yoshida & Tanaka, 2022); RvCAHS1‐3 are abundant proteins in the soluble fraction of tardigrade total lysate after heat treatment, and localize in the cytoplasm (Yamaguchi et al., 2012). The CAHS genes are widely conserved in the class Eutardigrada with multiple independent duplications within several lineages and intense expressions are observed in anhydrobiotic tardigrades (Fleming et al., 2023; Hashimoto et al., 2016; Yoshida et al., 2017). While the CAHS proteins possess high solubility under normal conditions, several reports have recently shown that CAHS proteins form a reversible higher order structure such as fibrous condensation and gel‐like structure at high concentration or under osmotic stress (Malki et al., 2021; Tanaka et al., 2022; Yagi‐Utsumi et al., 2021). In addition, a recently developed in vivo expression vector derived from the anhydrobiotic tardigrade genome, TardiVec, showed that the CAHS3 promoter mainly functions in the tardigrade epidermal cells (Tanaka et al., 2023). This finding suggests that anhydrobiosis‐related genes, including CAHS3, have a tissue‐specific expression to achieve anhydrobiosis with appropriate molecular mechanisms depending on each tissue; however, the regulation of tardigrade genes remains poorly understood.

The TardiVec system allows the experimental reconstitution of endogenous expression patterns in tardigrade cells; therefore, we tried to identify the regulatory sequence of the *CAHS3* gene. During the preconditioning period before entering anhydrobiosis, *H. exemplaris* induces around 1000 genes including CAHS family of proteins to successfully tolerate complete desiccation (Fleming et al., 2023; Hashimoto et al., 2016; Yoshida et al., 2017). Thus, identification of regulatory motifs likely realizes the identification of transcriptionally co‐regulated genes related to anhydrobiosis. Hence, here, we have approached this through both in vivo experiments and in silico analysis. Firstly, to explore the functional regulatory region of CAHS3, we stepwise cleaved the original 1 kbp upstream promoter region of TardiVec from the 5′ end. As a result, we found that approximately 300 to 350 bp upstream of the CAHS3 start codon is the key for expression of the downstream gene. In addition, we also found that further truncation of the CAHS3 promoter region affected the tissue specificity of the expression. Based on the experimental result, we explored potential motifs of regulatory elements in CAHS3 and further extended the exploration to genome‐wide upstream regions of *R. varieornatus* and *H. exemplaris*. The function of the motifs identified by in silico analysis was evaluated by motif deletion assays using the TardiVec system. One motif, MRv‐39, conserved between two tardigrade species, affected gene expression patterns with tissue specificity, suggesting that this motif could be widely used to express genes and regulate the tissue specificity in anhydrobiotic tardigrades.

## 2 RESULTS

### 2.1 In vivo screening with GFP expressing vectors highlighted a potential core promoter region of CAHS3

**FIGURE 1:**
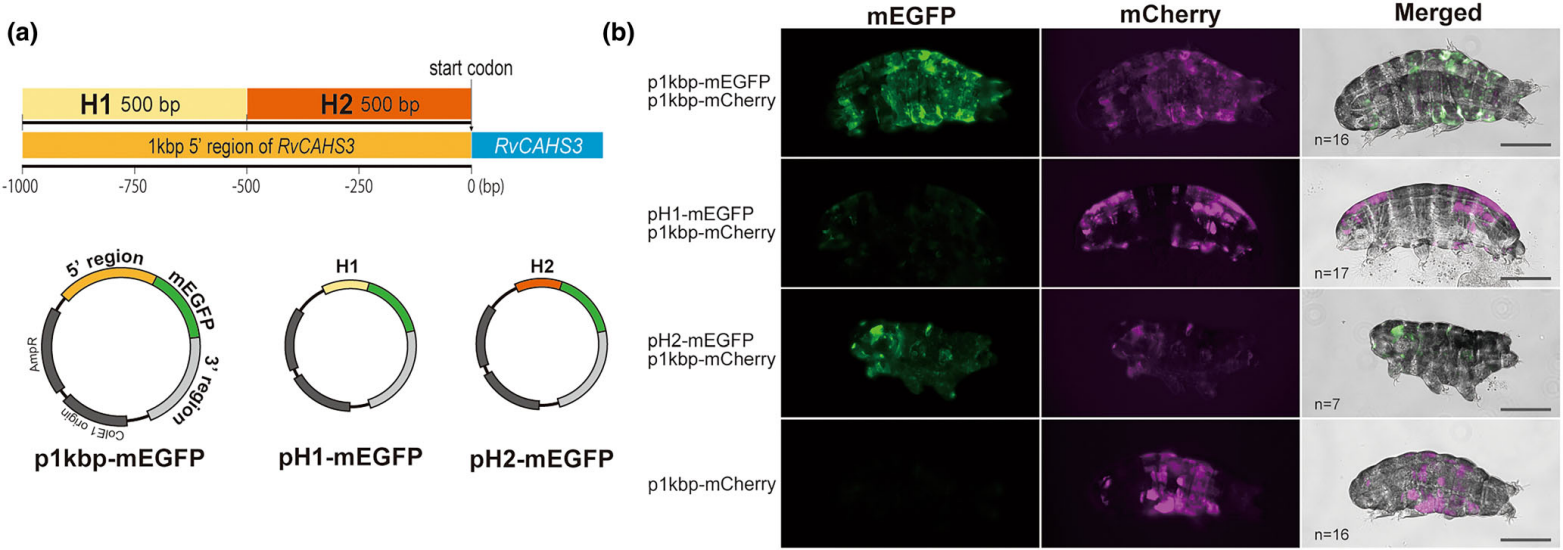
In vivo expression by half shortened promoter region of pRvCAHS3 vector in tardigrade. (a) Scheme of mEGFP‐expressing TardiVec in this study. Original TardiVec contains 1 kbp upstream of RvCAHS3 as the promoter region (p1kbp‐mEGFP). The half shortened vectors, the first (upstream, 5′) half, H1, and the second (downstream, 3′) half, H2, are derived from the −1000 to −500 bp, and −500 bp to 0 bp of the original vector, respectively. (b) Tardigrades, *R. varieornatus*, expressing mEGFP under the 1 kbp, first half and second half promoter sequence of RvCAHS3. mCherry was expressed under the 1 kbp promoter of RvCAHS3 (p1kbp‐mCherry) by co‐introduction with mEGFP vectors. mCherry expression is a control to show the expression pattern originating from the 1 kbp promoter. The merged images were obtained by overlaying two fluorescence images and bright‐field images. Sample Number is the number of animals that were observed to fluoresce. Scale bars: 100 μm.

Because the median length of intergenic regions is <1 kbp in the tardigrade genomes, Tardivec utilizes the 1 kbp upstream region of each gene, including CAHS3, to regulate the expression of inserted exogenous genes (Tanaka et al., 2023). However, it is not clear which of these regions are functional for gene expression. To explore the regulatory region, we first tested whether halving the 1 kbp upstream region retains gene expression in TardiVec (Figure 1a). As a result, the downstream half of the original 1 kbp promoter region, pRvCAHS3H2, showed a comparable GFP fluorescence to the original vector in transfected cells, while the upstream half, pRvCAHS3H1, emitted merely low fluorescence (Figure 1b). Moreover, fluorescence of mCherry, derived from a vector with the 1 kbp upstream region of CAHS3, was observed in the same epidermal cells as GFP expressed under pRvCAHS3H2 in cotransfected tardigrades. Therefore, the 500 bp upstream region of CAHS3 is sufficient for gene expression in the TardiVec system.

**FIGURE 2:**
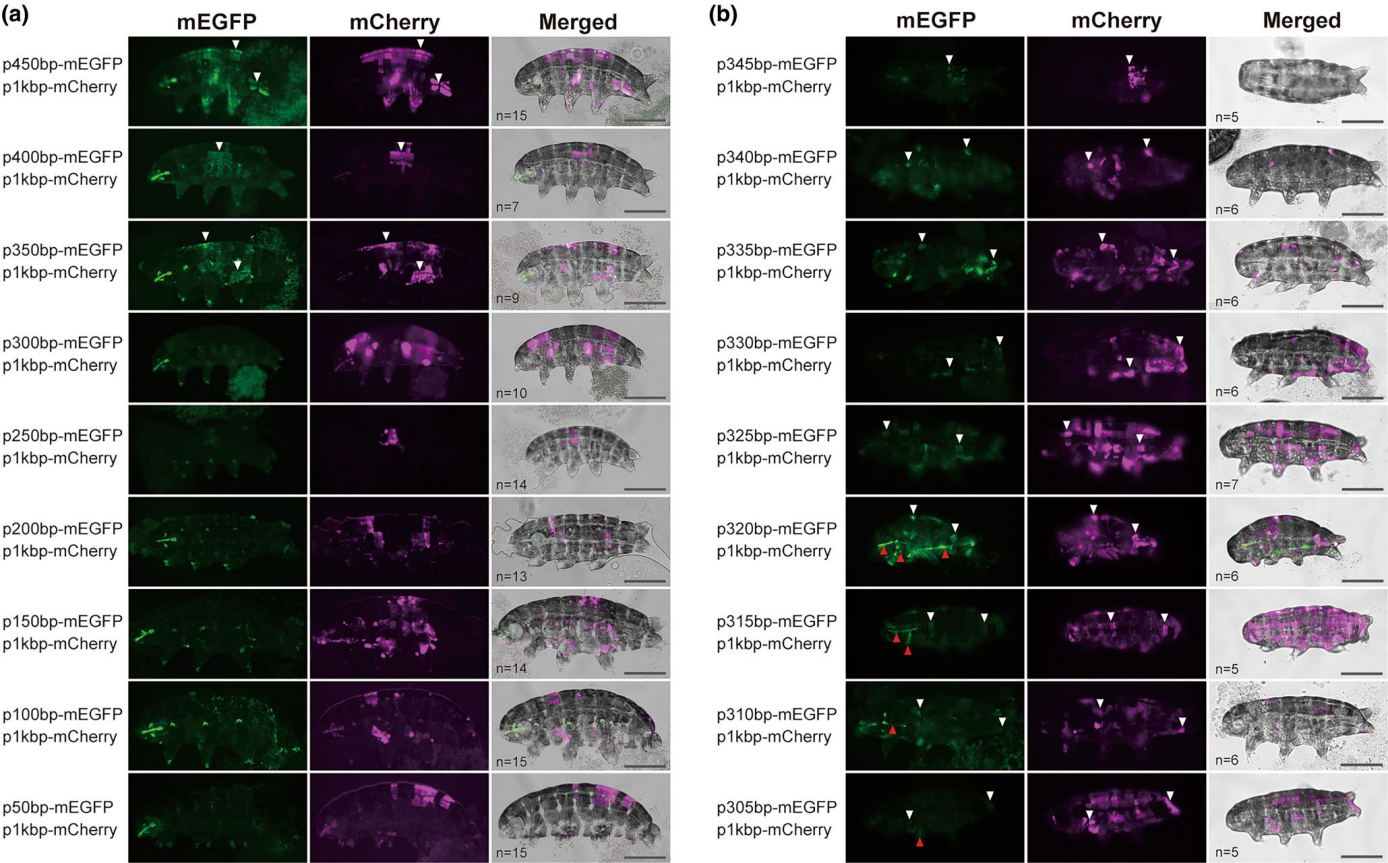
In vivo expression by step‐wised cleaved promoter region of pRvCAHS3 vector in tardigrade. (a) Tardigrades introduced vectors with 50 bp stepwise cleaved promoters from 450 to 50 bp. (b) Tardigrades introduced vectors with 5 bp stepwise cleaved promoters from 345 to 305 bp. mCherry was expressed under the 1 kbp promoter of RvCAHS3 (p1kbp‐mCherry) by co‐introduction with mEGFP vectors. mCherry expression is a control to show the expression pattern originating from the 1 kbp promoter. The merged images were obtained by superimposing two fluorescence and bright‐field images. White arrowheads indicate the epidermal cells where both mEGFP and mCherry were observed and red arrowheads indicate mEGFP signal in muscle cells. Scale bars: 100 μm.

Next, we examined the effect on gene expression of trimming the 5′ end of pRvCAHS3H2 in 50 bp increments (Figure 2a). Cutting to the 350 bp region still resulted in the specific expression in the epidermal cells, but the fluorescence intensity gradually became unstable, and GFP fluorescence was undetectable beyond 300 bp. The mCherry positive control expressed under intact 1 kbp upstream region was consistently observed predominantly in the epidermal cells, even when tardigrades showed no notable GFP fluorescence where they were cotransfected with vector in which the truncated 5′ region ranged from 300 to 50 bp. Therefore, the promoter functionality ceased at approximately 300 bp, suggesting that the functional promoter region of CAHS3 is located approximately 300 to 350 bp upstream of the start codon.

We then focused on the 300–350 bp region to search for the core promoter of CAHS3. When the length was decreased in 5 bp increments from 345 to 305 bp (Figure 2b), the intensity of GFP fluorescence decreased in epidermal cells transfected with these truncated vectors. In addition, interestingly, the tissue specificity of gene expression was distorted in the vectors with the promoter regions shorter than 320 bp. Expression of mEGFP in multiple muscle cells was observed in a few tardigrades (The proportions of individuals observed with this change were 2/6, 2/5, 2/6, and 2/5 at 320 bp, 315, 310, and 305 bp, respectively), while epidermal expression decreased. Strong fluorescence was observed predominantly in muscle cells with the 320 bp promoter length, whereas expression of mCherry under the 1 kbp promoter was not observed in any muscle cell, suggesting that there is a regulatory sequence of tissue specificity around 320 bp. The in vivo screening experiments indicate that 500 bp upstream of CAHS3 is sufficient for TardiVec gene expression and the 300–350 bp region upstream of the CAHS3 start codon is critical for regulating gene expression.

### 2.2 ab initio identification of conserved motifs in upstream of anhydrobiosis‐related genes

To understand the expression characteristics of CAHS3 in *R. varieornatus* prior to in silico analysis for gene regulatory elements, we examined the previously reported genomic and RNA‐seq data in the 10 kbp upstream and downstream regions of CAHS3 (Figure S1) (Hashimoto et al., 2016), because the putative Kozak sequence, analyzed using the G‐language Genome Analysis Environment v.1.9.2 (Arakawa et al., 2003), is poorly conserved in *R. varieornatus* (AANATGG is found in 1378 genes, but the conservation is quite low, 9.48% of the total genes; Figure S2). The closest neighboring genes to the CAHS3 gene are located 2.69 kbp upstream and 715 bp downstream. Among the genes in the 20 kbp region, one gene with known function was identified, namely GPR137B, a lysosomal integral membrane protein associated with the regulation of localization and activation of mTORC1 in *Mus musculus*, and no anhydrobiosis‐related gene was found. CAHS3 consists of five exons with high RNA‐seq coverage, and no splicing variant was identified. RNA reads were detected up to approximately 65 bp upstream of the CAHS3 start codon, indicating that this region is the 5′ UTR and suggested the potential transcription start site (TSS). Interestingly, at a much lower coverage, mapped upstream region sometime extended up to approximately 310 bp, which is close to the length where gene expression became defective when truncated in Tardivec expression, suggesting that this region is also tightly involved in CAHS3 gene expression on the tardigrade genome.

**FIGURE 3:**
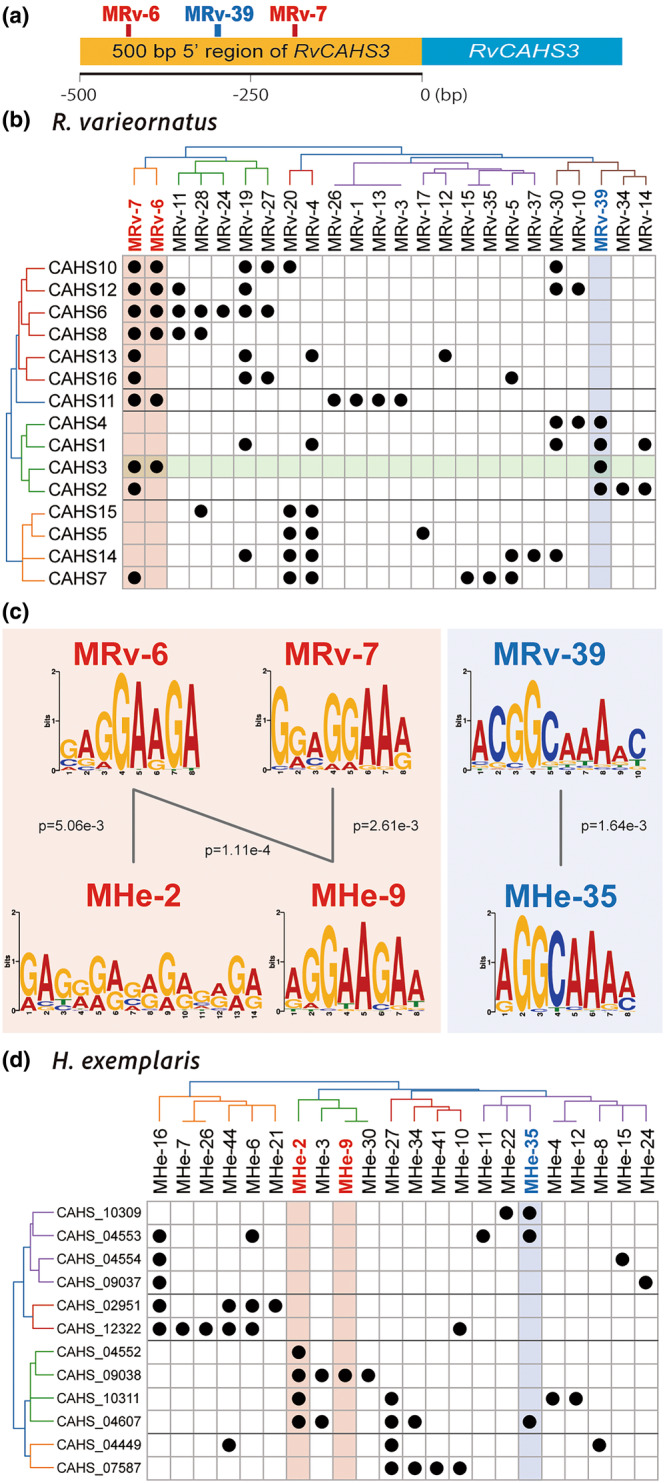
Putative motifs in the upstream region of the anhydrobiosis‐related gene family, *CAHS*, in two anhydrobiotic tardigrades. (a) Three putative motifs in the upstream region of RvCAHS3, identified through in silico analysis. (b) Conservation of motifs in CAHS gene family in *R. varieornatus*. (c) Motif sequence identified in RvCAHS3 and its similar motifs from *H. exemplaris*. (d) Conservation of motifs in CAHS gene family in *H. exemplaris*.

To further analyze the upstream sequence of CAHS3 in silico, we performed an ab initio exploration of motifs in 500 bp upstream sequences of all genes in *R. varieornatus* using STREME, an algorithm that can discover ungapped motifs de novo in large sequence datasets, because search for known motifs such as TATA‐box did not succeed in this distinct phylum with the PWMscan program (Ambrosini et al., 2018). As a result, a total of 39 motifs were discovered, and specifically, in the upstream sequence of CAHS3, three motifs, MRv‐6, MRv‐7, and MRv‐39, were significantly identified (Figure 3a and Table S1). MRv‐6 GAGGAAGA and MRv‐7 GGAGGAAR comprises approximately 50% of all identified motif sites (3560 and 4035 sites, respectively), suggesting that these motifs are widely used in the genome. MRv‐6 was located on the negative strand 440 to 433 bp relative to the start codon, and MRv‐7 was on the negative strand 191 to 184 bp. MRv‐39 ACGGCAAAAC was identified at a total of 1032 sites (12.9%) and was located on the negative strand from 307 to 298 bp, close to a region experimentally identified as important for expression, suggesting that this motif may be a good candidate for a regulatory element. To verify the effect of repetitive elements in de novo identification by STREME, we modeled tardigrade‐unique repetitive elements using RepeatModeler (Smit and Hubley 2008–2015), which revealed 895 sequences that appear cumulatively 38,253 times in the tardigrade genome, comprising 12.12% of its genome. In addition, Repbase‐based searches for repetitive elements and simple repeats using RepeatMasker (Smit et al., 2004) identified 3.56% and 0.22% of repetitive elements, respectively, totaling 15.90% of the genome in *R. varieornatus*. The amounts of repetitive elements are remarkably low in this genome, reflecting the compact nature of the 55Mbp genome. Consequently, none of the MRv‐6, MRv‐7, and MRv‐39 identified in the upstream region of CAHS3 correspond to these repetitive sequences, so we believe that our results are robust to the presence of repetitive elements within the genome.

Next, we performed cluster analysis on the ab initio motifs identified 500 bp upstream of the CAHS gene family to investigate motif conservation (Figure 3b). MRv‐6 and MRv‐7 were also highly conserved in the CAHS gene family, with 10 out of 15 having at least one of the motifs in their upstream. When the cluster analysis was extended to the anhydrobiosis‐related genes, more than half of the genes contained these motifs (Figure S3). On the other hand, MRv‐39 was found in four CAHS genes and in 10 anhydrobiosis‐related genes (14.5% of all anhydrobiosis‐related genes); CAHS1‐4, SAHS6, SAHS11 and five paralogs of AMNP (Yoshida et al., 2022, 2024). Among them, it is interesting to note that CAHS1 and CAHS2, as well as CAHS3, were initially identified as abundant heat‐soluble proteins in the tardigrade lysate. In addition to MRv‐39, the upstream of CAHS2 also contains MRv‐7 and is therefore clustered near CAHS3, while CAHS1 shares MRv‐14 with CAHS2, suggesting that these regulatory motifs may be related to regulate the abundant expression of anhydrobiosis‐related genes.

To understand the inter‐specific conservation of the regulatory element of tardigrade anhydrobiosis‐related genes, further analysis was conducted in the 500 bp upstream regions of all *H. exemplaris* genes, resulting in a total of 49 motifs (Table S1). Motif comparison between the two species revealed that 87.8% of the motifs shared at least one significantly similar counterpart, and 44.9% were bidirectionally significant (significance *p* value threshold at .05). In addition, 64 of *R. varieornatus* genes and 55 of *H. exemplaris* genes were identified to have significant counterparts among tardigrade‐specific anhydrobiosis‐related genes. In particular, MRv‐39 and MHe‐35 matched significantly, and MRv‐6 and MRv‐7 also had significant bidirectional similarity to MHe‐2 and MHe‐9 (Figure 3c). MHe‐35 was detected in a total of 14 anhydrobiosis‐related genes, including three CAHS family genes, five SAHS family genes, and six AMNP family genes in *H. exemplaris* (Figure 3d and S4). Genes with MHe‐35 in their upstream were detected in several clusters and across different types of anhydrobiosis‐related genes, as was MRv‐39 in *R. varieornatus*, although both MRv‐39 and MHe‐35 were rarely found among conserved homologs, except for one AMNP (g244 and BV898_04755). On the other hand, MHe‐2 and MHe‐9 were detected in only 18 and 13 out of 55 genes (32.7% and 23.6%), and the prevalence is low compared to widely detected MRv‐6 and MRv‐7, suggesting a more complex promoter orientation and regulatory system.

**FIGURE 4:**
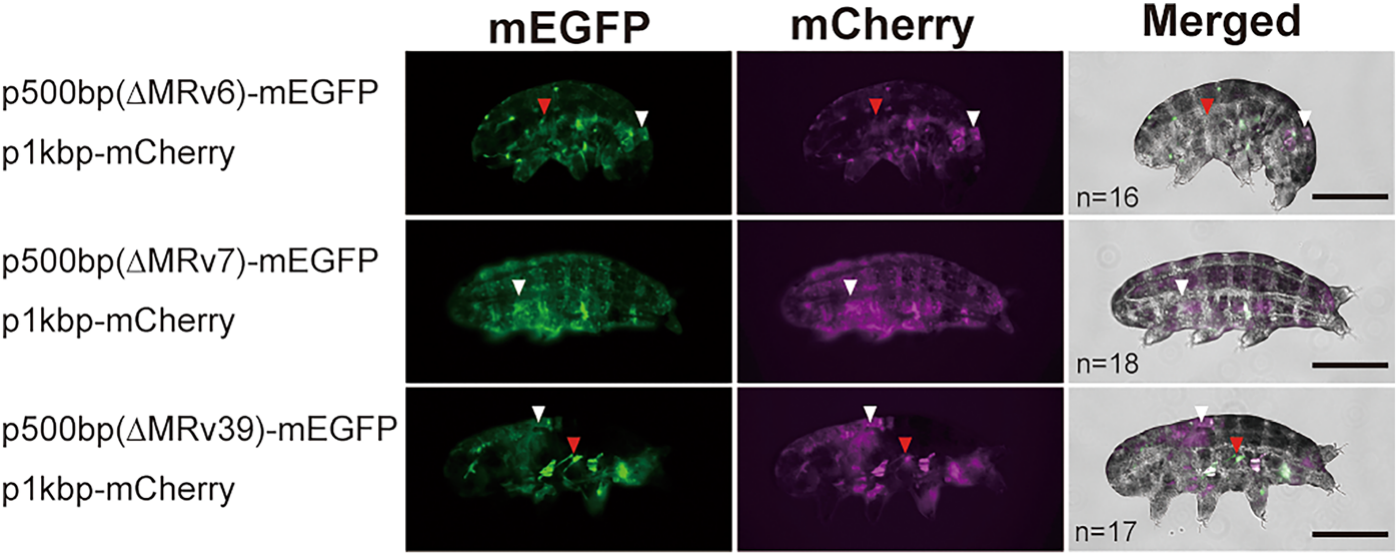
In vivo expression by deletion vectors of each putative motif in tardigrades. Tardigrades introduced vectors with 500 bp upstream region that were deleted at each putative motif, MRv6, MRv7, and MRv39. mCherry was expressed under the 1 kbp promoter of RvCAHS3 (p1kbp‐mCherry) by co‐introduction with mEGFP vectors. The merged images were obtained by superimposing two fluorescence and bright‐field images. White arrowheads indicate the epidermal cells where both mEGFP and mCherry were observed and red arrowheads indicate mEGFP signal in muscle cells. Scale bars: 100 μm.

To experimentally test the effect of each motif on transcription, we examined deletion mutants of MRv‐39, MRv‐6, and MRv‐7 motifs (Figure 4). Although no significant loss of fluorescence was observed for any deletion, interestingly, ∆MRv‐39 showed a change in tissue‐specific expression with bright fluorescence showing up in muscles in addition to epidermis, and ∆MRv‐6 also showed weak fluorescence in muscle (this was observed for 5 out of 17 animals with ΔMRv‐39 and 4 out of 16 animals with ΔMRv‐6). The result suggests that MRv‐39, which is also found in CAHS1 that is mainly expressed in epidermis and not in muscle, may act as a suppressor of expression in muscle. Consequently, our de novo in silico exploration of motif sequences in the tardigrade genome successfully discovered promising candidate motifs thereof, possibly related to the regulation of transcription of anhydrobiosis‐related genes.

## 3 DISCUSSION

Our present study has shown that 500 bp upstream of *CAHS3* is sufficient and the 300–350 bp upstream is critical for gene expression on the TardiVec system. Moreover, by focusing on the 500 bp upstream of all genes based on this finding, we have identified several motif sequences, such as MRv‐6, MRv‐7, and MRv‐39, that are conserved in the upstream sequence of anhydrobiotic‐related genes in two species of anhydrobiotic tardigrades, *R. varieornatus and H. exemplaris*. The possibility that the regulatory motifs are conserved in Eutardigrada, including these two tardigrades, was suggested in the previous study based on the finding that the promoter sequence of *R. varieornatus CAHS3* allows heterologous expression in *H. exemplaris, Thulinius ruffoi*, and *Milnesium inceptum* (Tanaka et al., 2023). These tardigrade species appear to have conserved systems that regulate when, where, and how much genes are expressed, even though they differ in their ability to enter an anhydrous state. This is further supported by the finding that similar motifs were independently identified with ab initio in silico methods in two different genomes.

Three motifs were identified in the 500 bp upstream of RvCAHS3 that are also conserved in *H. exemplaris*. A search for similarity to known transcription factor (TF) binding motifs against MRv‐39 identified TF class C2H2 zinc cluster factors and C6 zinc cluster factors, although they did not show significant *e*‐values (Fedotova et al., 2017; Gupta et al., 2007; Han et al., 2020), while MHe‐35 showed similarity to different types of TFs, C4 zinc finger‐type factor (Brown, 2005) (Table S2). On the other hand, MRv‐6 and MRv‐7 showed similarity with C2H2 zinc finger factors and tryptophan cluster factors, as did MHe‐2 and MHe‐9. The deletion of MRv‐39 showed disrupted tissue‐specific expression with bright fluorescence in muscle in addition to epidermis. Moreover, the 5 bp deletion mutant, ∆325–320 bp, showed gene expression in muscle likewise ∆MRv‐39 (Figure S5), suggesting that the region around 325–300 bp possibly function to suppress gene expression in muscle in tardigrades. In addition, the finding that these distinct deletion mutants exhibiting similar consequences may also suggest a functional association between them. We could not identify any motif in the 325–320 bp region by in silico analysis; however, in the future, ATAC‐seq analysis (Buenrostro et al., 2015), which can identify open chromatin regions, performed in each cell type, tissue and condition of tardigrades would provide informative data to narrow down the target regions. Further narrowing of the analysis is likely to discover additional motifs unique to tardigrades that regulate anhydrobiosis‐related gene expression. Quantitative discussion related to gene expression is challenging at the current individual‐level analysis; nevertheless, other motifs identified in this study, such as MRv‐6 and MRv‐7, also seem to contribute to the regulation of transcriptional activity or repression in anhydrobiotic genes as suggested by the results of deletion mutants of these motifs.

Most of the tardigrade anhydrobiosis‐related genes that have been identified so far are unstructured proteins that are unique to tardigrades (Arakawa, 2022; Hashimoto et al., 2016; Tanaka et al., 2015; Yamaguchi et al., 2012; Yoshida & Tanaka, 2022). These nondomain proteins tend to have lower amino acid sequence conservation compared to structured proteins such as enzymes (Arakawa et al., 2023), and their protein function remains mostly elusive; a domain that forms a fiber‐like structure has been identified in CAHS3 although this feature does not seem to be conserved among the CAHS family in Eutardigrada (Fleming et al., 2023; Tanaka et al., 2022). Furthermore, previous transcriptome analyses have shown that the expression levels of the CAHS paralogs varies widely even in adult tardigrades from one to four orders of magnitude, and that several CAHS paralogs are mainly expressed in embryos (Yoshida et al., 2017, 2019). Development of the TardiVec system realizing live imaging in tardigrades in vivo elucidated surprising tissue specificity of tardigrade‐unique genes, but the tissue specificity of CAHS paralogs is yet to be identified (Tanaka et al., 2023). In the present study, we attempted to classify the CAHS gene family based on the regulation mechanism of their gene expression by focusing on the motif conservation between CAHS paralogs. As a result, the CAHS gene family was classified into three or four cluster groups according to the conservation pattern of upstream motifs. Interestingly, the clustering seems to somewhat reflect the expression levels. The CAHS1‐4 cluster contains CAHS1‐3 that are originally identified as abundant proteins in the heat‐soluble fraction of *R. varieornatus*. On the other hand, another cluster consisted of CAHS genes with low expression levels, namely, CAHS5, 7, 14 and 15. CAHS6 and 8 in the remaining cluster are the two paralogs most significantly induced in the embryo. These observations altogether provide supporting evidence as to when, where, and how much the genes are expressed in the tardigrade, and extrapolating this knowledge across other tardigrade‐unique genes of unknown function may help elucidate the birds eye view of the comprehensive anhydrobiosis machinery.

The ability to perform live imaging in tardigrades using TardiVec is bringing new developments to the field of molecular anhydrobiology, and the possibility in pinpointing the gene regulatory motifs as shown in this study is one of such applications. Furthermore, the findings of this study that the functional promoter region of highly expressed CAHS3 can be halved down to 500 bp synergistically enhances the utility of TardiVec, for smaller vectors are easier to prepare and potentially more readily delivered into tardigrade cells. In addition, for an experiment that does not require tissue‐specific expression, an even smaller vector with a 320 bp promoter can be used. Although the 3′ region was not examined in this study, it is believed that it is possible to further reduce the size of the vector likewise in the downstream transcription termination region. Further studies will require comprehensive analysis such as Hi‐C analysis for more long range interactions (Belton et al., 2012) and ChiP‐seq (Furey, 2012) or DAP‐seq (Bartlett et al., 2017) to pinpoint TF‐promoter interaction, and the TardiVec system could validate the proximal regulatory sequence in each tardigrade tissue in combination with such methods. Understanding the evolution of diverse regulatory mechanisms and multiple gene duplications in anhydrobiotic tardigrades would lead to the elucidation of the molecular mechanisms of anhydrobiosis as well as to the methods to modify and regulate the machinery.

## 4 EXPERIMENTAL PROCEDURES

### 4.1 In vivo screening of promoter region

A series of stepwise truncated upstream regions of RvCAHS3 and the remaining part of pRvCAHS3‐mEGFP were separately amplified with designed primers using Tks Gflex DNA Polymerase (Takara Bio Inc.). These two PCR fragments were assembled into a circular DNA plasmid using NEBuilder HiFi DNA Assembly Master Mix (New England BioLabs, Inc.). The assembled plasmids were introduced into DH5α cells and purified using Plasmid Plus Midi Kit (QIAGEN). All of the plasmid solutions were adjusted to 4 μg/μl. pRvCAHS3‐mCherry (mCherry expression vector containing 1 kbp promoter region of RvCAHS3) was used as a positive control for the introduction of TardiVec into a tardigrade cell, and injection plasmid solutions were prepared with a mixture of pRvCAHS3‐mCherry and each truncated promoter vector expressing mEGFP. *R. varieornatus* (YOKOZUNA‐1) which were reared on a 2% agar plate at 22°C by feeding on Chlorella V‐12 (Chlorella Industry Co., Ltd.) were used for microinjection experiments.

Plasmid DNA injection experiments were conducted according to the method in the previous study (Tanaka et al., 2023). Tardigrades were injected the plasmid mixture using an IM‐400 Electric Microinjector (NARISHIGE Group) attached to an Axio Vert.A1 inverted microscope (ZEISS) and electroporated by the Super Electroporator NEPA21 Type II (NEPA GENE Co., Ltd.) (125 V, pulse length: 5 ms, 3 pulses, decay rate: 10%, polarity: +/−). EVOS M5000 Imaging System (ThermoFisher Scientific, Inc.) was used to observe the treated tardigrades after 48 h. Fluorescence of mEGFP was observed under light cube AMEP4951 (excitation 470/22 nm, luminescence 525/50 nm), and mCherry was observed under light cube AMEP4952 (excitation 531/40 nm, luminescence 593/40 nm).

### 4.2 Ab initio motif exploration in the upstream of tardigrade genes

Within the datasets of the 500 bp upstream sequences of all genes in *R. varieornatus* and *H. exemplaris*, ab initio motif exploration was performed using STREME (Sensitive, Thorough, Rapid, Enriched Motif Elicitation) version 5.5.5 (Bailey, 2021). Motif significance thresholds were set at *p* value .05. Each significant motif was then compared to the core promoter database, JASPAR CORE 2022 (Castro‐Mondragon & Riudavets‐Puig, 2022), using the Motif Comparison Tool, Tomtom version 5.5.5, to identify similar motifs (Gupta et al., 2007). Motifs were compared between anhydrobiosis‐related genes identified in both tardigrade species to examine motif conservation within the upstream regions of these genes. Identified motifs were clustered according to the genes to which they were related, and genes were clustered according to motif conservation. Clustering was performed using a custom R script.

## Supporting information


**FIGURE S1.** RNA‐seq reads and coverage of RvCHAS3. (a) RNA reads and coverage in 10 kbp upstream and downstream of CAHS3 (Scaffold 13: 649,624 ~ 670,909 bp). Each gene is represented with specific orientation using arrows. Gray arrows represent hypothetical proteins. GPR137B is an ortholog of an integral membrane protein associated with the regulation of localization and activity of mTORC1 in *M. musculus*. Red and Blue reads each represent reads from the positive strand and negative strand. (b) RNA read and coverage of RvCHAS3 and its 1 kbp upstream and downstream regions. CAHS3 gene is oriented on the negative strand with five exon regions. Black arrows at the top indicate the start codon (right) and stop codon (left) of CAHS3. Reads on the left show the gene region of g3883 (hypothetical protein). White arrows indicate possible TSS location −65 and −312 bp relative to the start codon. Locations for MRv‐7 and MRv‐6 are highlighted in gray. MRv‐39 is highlighted in pink. The pair‐end RNA‐seq data of active adults of *R. varieornatus* was downloaded via the following accession (DRR013911) (Fleming et al., 2023; Hashimoto et al., 2016; Yoshida et al., 2017).
**FIGURE S2.** Putative Kozak sequence in *R. varieornatus*. Consensus sequence AANATGG is found in start codon of 1378 genes, 9.48% of the total genes.
**FIGURE S3.** Motifs identified in known anhydrobiosis‐related genes in *R. varieornatus*. The comprehensive match of *R. varieornatus* anhydrobiosis‐related genes with motif conservation are plotted.
**FIGURE S4.** Motifs identified in known anhydrobiosis‐related genes of *H. exemplaris*. The comprehensive match of *H. exemplaris* anhydrobiosis‐related genes with motif conservation are plotted.
**FIGURE S5.** In vivo expression by 5 bp deletion mutant, ∆325–320 bp, in tardigrades. Tardigrades introduced vectors with 500 bp upstream region that was deleted at 325–320 bp. mCherry was expressed under the 1 kbp promoter of RvCAHS3 (p1kbp‐mCherry) by co‐introduction with mEGFP vectors. The merged images were obtained by superimposing two fluorescence and bright‐field images. White arrowheads indicate the epidermal cells where both mEGFP and mCherry were observed and red arrowheads indicate mEGFP signal in muscle cells. Scale bars: 100 μm.
**TABLE S1.** Motifs significantly identified in the 500 bp upstream region of all genes in *R. varieornatus* and *H. exemplaris*.
**TABLE S2.** Motif similarity to TF binding site database.
