# Supplementary material for: Search for putative gene regulatory motifs in CAHS3, linked to anhydrobiosis in a tardigrade Ramazzottius varieornatus, in vivo and in silico
Source: Genes Cells. 2024 Sep 30;29(12):1144–53. doi: 10.1111/gtc.13168 (PMC11609603; doi:10.1111/gtc.13168)
Supplement: Supplementary file 1 — FIGURE S1. RNA‐seq reads and coverage of RvCHAS3. (a) RNA reads and coverage in 10 kbp upstream and downstream of CAHS3 (Scaffold 13: 649,624 ~ 670,909 bp). Each gene is represented with specific orientation using arrows. Gray arrows represent hypothetical proteins. GPR137B is an ortholog of an integral membrane protein associated with the regulation of localization and activity of mTORC1 in M. musculus. Red and Blue reads each represent reads from the positive strand and negative strand. (b) RNA read and coverage of RvCHAS3 and its 1 kbp upstream and downstream regions. CAHS3 gene is oriented on the negative strand with five exon regions. Black arrows at the top indicate the start codon (right) and stop codon (left) of CAHS3. Reads on the left show the gene region of g3883 (hypothetical protein). White arrows indicate possible TSS location −65 and −312 bp relative to the start codon. Locations for MRv‐7 and MRv‐6 are highlighted in gray. MRv‐39 is highlighted in pink. The pair‐end RNA‐seq data of active adults of R. varieornatus was downloaded via the following accession (DRR013911) (Fleming et al., 2023; Hashimoto et al., 2016; Yoshida et al., 2017). FIGURE S2. Putative Kozak sequence in R. varieornatus. Consensus sequence AANATGG is found in start codon of 1378 genes, 9.48% of the total genes. FIGURE S3. Motifs identified in known anhydrobiosis‐related genes in R. varieornatus. The comprehensive match of R. varieornatus anhydrobiosis‐related genes with motif conservation are plotted. FIGURE S4. Motifs identified in known anhydrobiosis‐related genes of H. exemplaris. The comprehensive match of H. exemplaris anhydrobiosis‐related genes with motif conservation are plotted. FIGURE S5. In vivo expression by 5 bp deletion mutant, ∆325–320 bp, in tardigrades. Tardigrades introduced vectors with 500 bp upstream region that was deleted at 325–320 bp. mCherry was expressed under the 1 kbp promoter of RvCAHS3 (p1kbp‐mCherry) by co‐introduction with mEGFP vectors. The merged [file GTC-29-1144-s001.pdf]

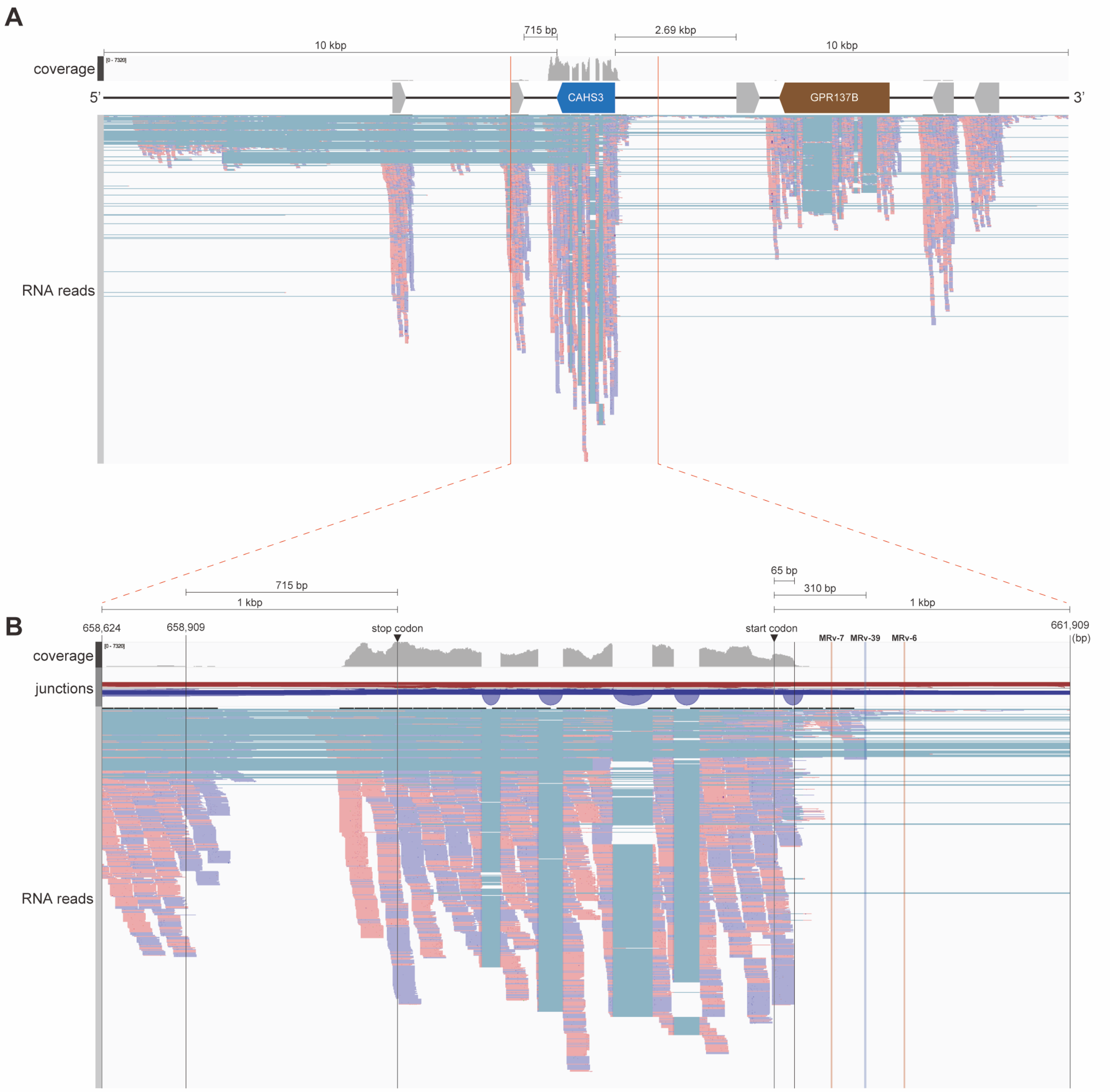

Figure S1 RNA-seq reads and coverage of RvCHAS3

(A) RNA reads and coverage in 10 kbp upstream and downstream of CAHS3 (Scaffold 13: 649,624 ~ 670,909 bp). Each gene is represented with specific orientation using arrows. Gray arrows represent hypothetical proteins. GPR137B is an ortholog of an integral membrane protein associated with the regulation of localization and activity of mTORC1 in *M. musculus*. Red and Blue reads each represent reads from the positive strand and negative strand. (B) RNA read and coverage of RvCHAS3 and its 1 kbp upstream and downstream regions. CAHS3 gene is oriented on the negative strand with five exon regions. Black arrows at the top indicate the start codon (right) and stop codon (left) of CAHS3. Reads on the left show the gene region of g3883 (hypothetical protein). White arrows indicate possible TSS location -65 bp and -312 bp relative to the start codon. Locations for MRv-7 and MRv-6 are highlighted in gray. MRv-39 is highlighted in pink. The pair-end RNA-seq data of active adults of *R. varieornatus* was downloaded via the following accession (DRR013911) (Hashimoto et al., 2016).

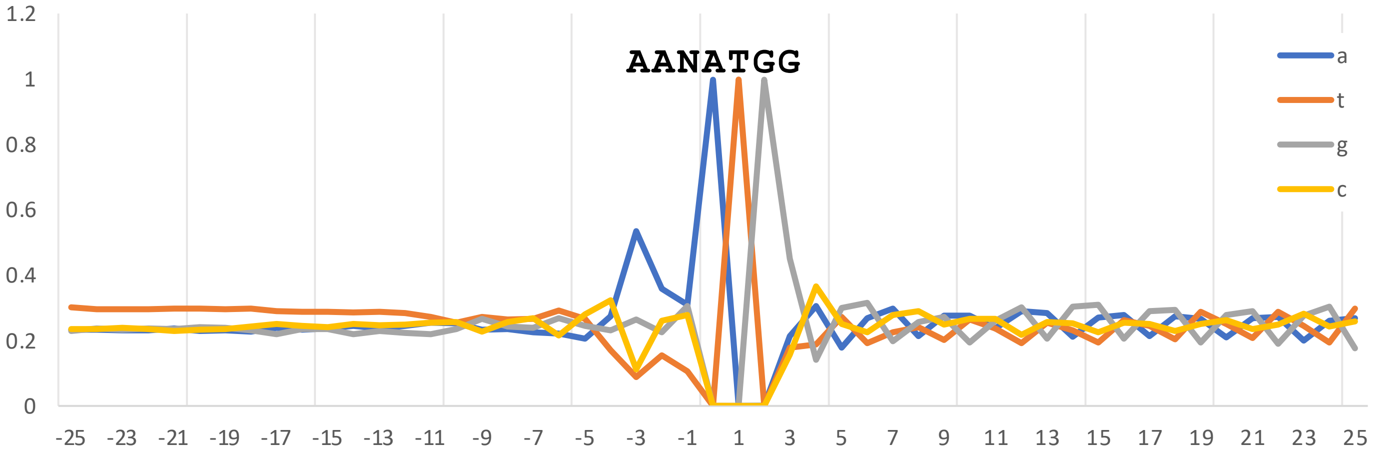

Figure S2 Putative Kozak sequence in *R. varieornatus*

Consensus sequence AANATGG is found in start codon of 1,378 genes, 9.48% of the total genes.

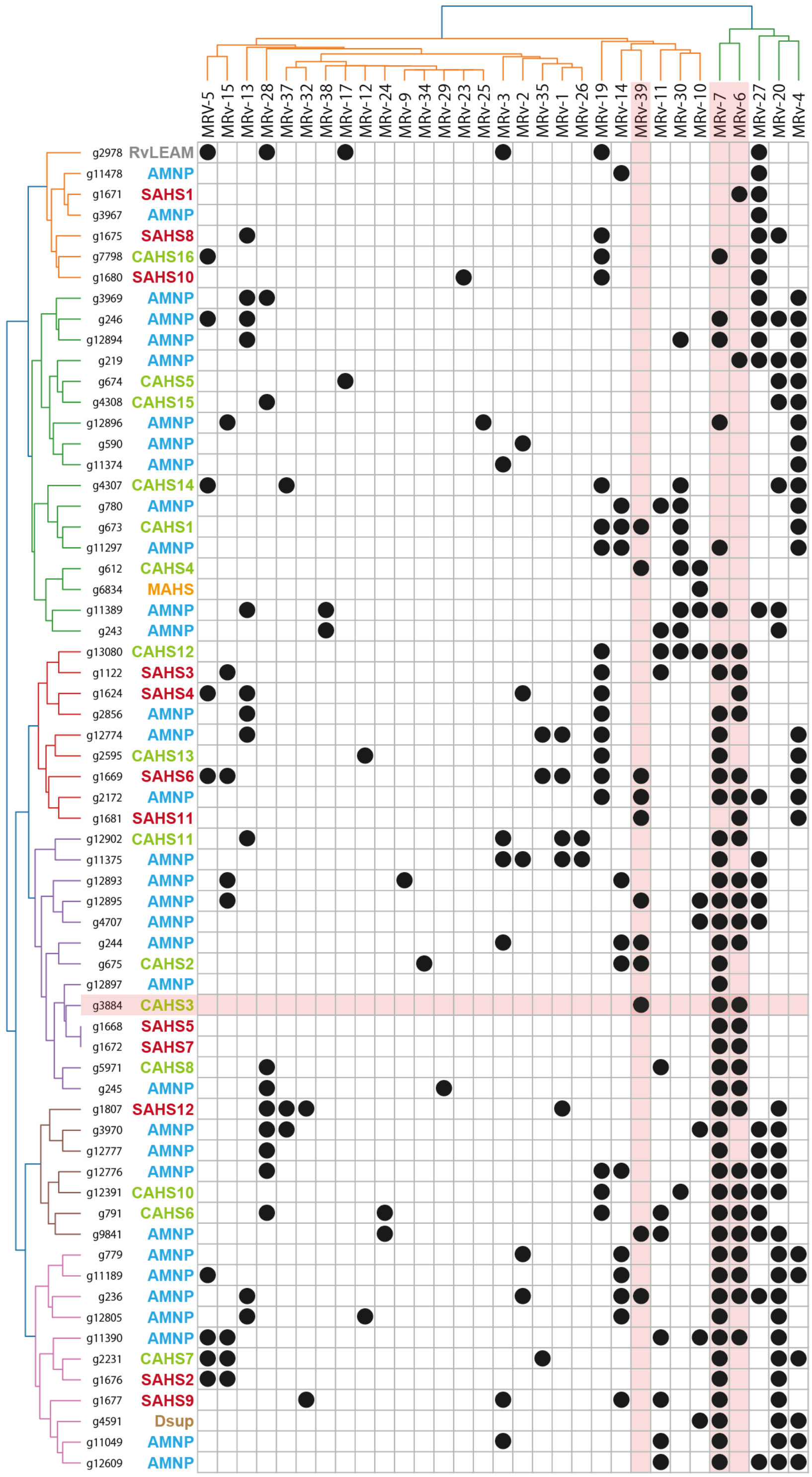

Figure S3 Motifs identified in known anhydrobiosis-related genes in *R. varieornatus*

The comprehensive match of *R. varieornatus* anhydrobiosis-related genes with motif conservation are plotted.

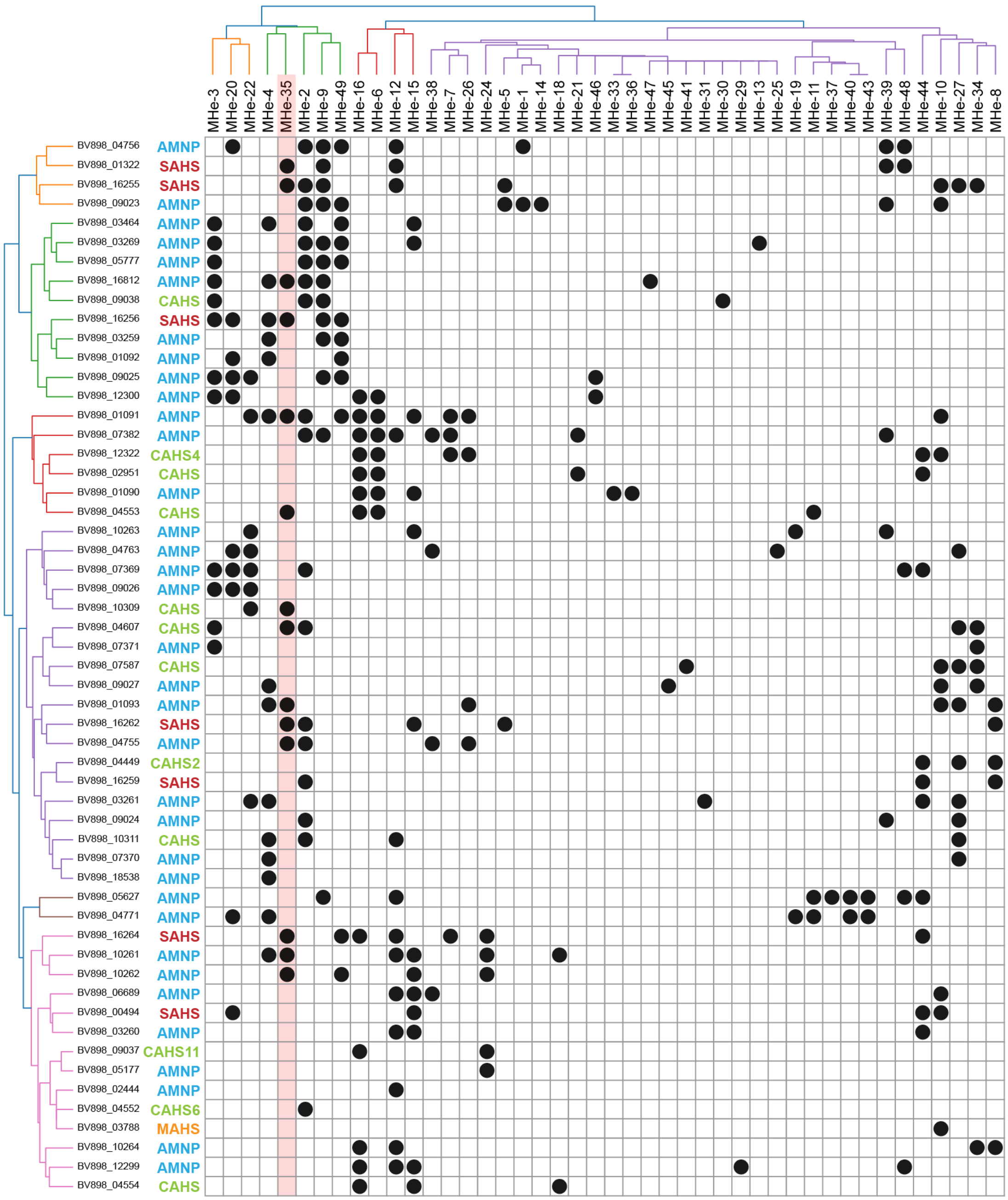

Figure S4 Motifs identified in known anhydrobiosis-related genes of *H. exemplaris*

The comprehensive match of *H. exemplaris* anhydrobiosis-related genes with motif conservation are plotted.

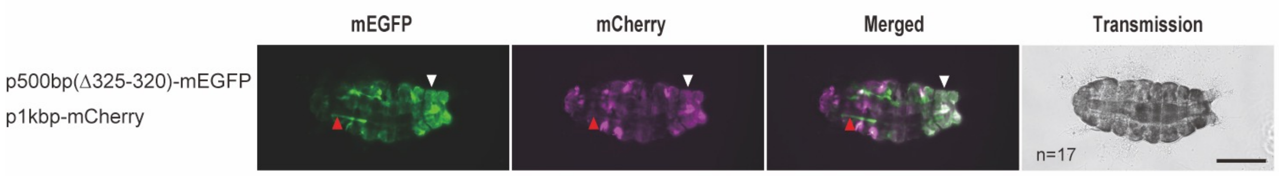

Figure S5 *in vivo* expression by 5bp deletion mutant, Δ325-320 bp, in tardigrades
Tardigrades introduced vectors with 500 bp upstream region that was deleted at 325-320 bp. mCherry was expressed under the 1 kbp promoter of RvCAHS3 (p1kbp-mCherry) by co-introduction with mEGFP vectors. The merged images were obtained by superimposing two fluorescence and bright-field images. White arrowheads indicate the epidermal cells where both mEGFP and mCherry were observed and red arrowheads indicate mEGFP signal in muscle cells. Scale bars: 100 μm.

Table S1 Motifs significantly identified in the 500 bp upstream region of all genes in *R. varieornatus* and *H. exemplaris*

Motifs in *R. varieornatus*		P-value	Sites
MRv-1	KVTTGATTGAYTRWT	1.20E-21	1872 (23.4%)
MRv-2	ACCGGAAGTSVVN	6.80E-16	919 (11.5%)
MRv-3	ATGACGTCACVNN	5.00E-12	1109 (13.9%)
MRv-4	WWAATTWW	2.20E-09	3848 (48.1%)
MRv-5	WVTACWGTABW	8.70E-09	1634 (20.4%)
MRv-6	GAGGAAGA	6.40E-08	3560 (44.5%)
MRv-7	GGAGGAAR	2.20E-07	4035 (50.4%)
MRv-8	AGTGGCGCACD	3.80E-07	312 (3.9%)
MRv-9	ATCGCAATCTCKM	1.40E-06	242 (3.0%)
MRv-10	AAAACSAMAACAA	3.80E-06	1059 (13.2%)
MRv-11	ACCTGCAAM	6.70E-06	1630 (20.4%)
MRv-12	AWCAGCTGATC	9.00E-06	445 (5.6%)
MRv-13	CAATCAACC	1.80E-04	791 (9.9%)
MRv-14	GAAATTTC	6.80E-04	1683 (21.0%)
MRv-15	CTGGAAGA	8.70E-04	1077 (13.5%)
MRv-16	TCCAGAHTCCAGA	8.80E-04	120 (1.5%)
MRv-17	TACGTCAYYG	1.30E-03	294 (3.7%)
MRv-18	AGCAGCWCATCAGTG	1.90E-03	88 (1.1%)
MRv-19	AAGATGGCS	2.10E-03	2369 (29.6%)
MRv-20	AAAATCAA	2.80E-03	2234 (27.9%)
MRv-21	AGCGGTGGACGGGTG	3.80E-03	76 (0.9%)
MRv-22	ATGACATTA	3.80E-03	96 (1.2%)
MRv-23	GATGGATGGAG	5.70E-03	160 (2.0%)
MRv-24	GAAAGTYGARAC	5.90E-03	245 (3.1%)
MRv-25	CTTCCGTTTCCG	6.30E-03	224 (2.8%)
MRv-26	GCYCCGCCC	7.60E-03	291 (3.6%)
MRv-27	CTCGACCA	1.00E-02	2634 (32.9%)
MRv-28	CATCATCCAC	1.00E-02	1185 (14.8%)
MRv-29	CGAGGGGATTWTGC	1.50E-02	60 (0.8%)
MRv-30	AGWAAACT	2.90E-02	966 (12.1%)
MRv-31	ATGGCAGGCC	3.10E-02	216 (2.7%)
MRv-32	ATGTTAAAAAGCC	3.10E-02	49 (0.6%)
MRv-33	ATCGTATCGAAAT	3.10E-02	41 (0.5%)
MRv-34	CCACTGGTCA	3.10E-02	46 (0.6%)
MRv-35	ATAWWWTAT	3.50E-02	90 (1.1%)
MRv-36	CCTAATATCA	3.50E-02	65 (0.8%)
MRv-37	GTACACTGTA	3.80E-02	178 (2.2%)
MRv-38	CAGATGGCGC	4.50E-02	137 (1.7%)
MRv-39	ACGGCAAAAC	5.00E-02	1032 (12.9%)

Motifs in *H. exemplaris*		P-value	Sites
MHe-1	AATCAATCAATCAAT	3.90E-24	1241 (15.5%)
MHe-2	GAGRGRGRGRGRGA	3.50E-21	3122 (39.0%)
MHe-3	CAGACASACASACA	1.50E-14	1040 (13.0%)
MHe-4	ACCGGAAG	2.50E-08	1526 (19.1%)
MHe-5	RTTATTATTAYT	8.00E-07	820 (10.3%)
MHe-6	WSTACWGTASW	3.30E-06	676 (8.4%)
MHe-7	GCCCCGCCYCC	3.40E-06	433 (5.4%)
MHe-8	AAACCACAACAAA	1.10E-05	421 (5.3%)
MHe-9	AGGAAGAA	2.30E-05	2417 (30.2%)
MHe-10	TACHDGTA	2.30E-05	733 (9.2%)
MHe-11	ACCGAGTTAAGCCAT	6.70E-05	197 (2.5%)
MHe-12	AAWATAWTT	1.00E-04	1793 (22.4%)
MHe-13	VAWCAGCTGWTB	1.40E-04	301 (3.8%)
MHe-14	CCTTGAGATCGSGAG	2.30E-04	96 (1.2%)
MHe-15	AAAATAAAA	3.00E-04	2360 (29.5%)
MHe-16	DTACNGTAH	4.20E-04	765 (9.6%)
MHe-17	CGACGACGAC	4.60E-04	132 (1.6%)
MHe-18	TATATATATATATATA	7.50E-04	466 (5.8%)
MHe-19	ACACACGTGGCMAAA	8.80E-04	214 (2.7%)
MHe-20	ACACACACAC	1.40E-03	716 (8.9%)
MHe-21	TCATCATCAT	2.10E-03	719 (9.0%)
MHe-22	CCCATCCCY	2.60E-03	1141 (14.3%)
MHe-23	GTCAGTCA	2.90E-03	291 (3.6%)
MHe-24	CAGCAGCAGC	3.30E-03	510 (6.4%)
MHe-25	AGCGCCACCT	3.40E-03	190 (2.4%)
MHe-26	NRTGACGTCAYN	3.40E-03	220 (2.8%)
MHe-27	GAGATGGAA	4.20E-03	1190 (14.9%)
MHe-28	AGAGGTCGCTGA	7.70E-03	67 (0.8%)
MHe-29	CGGCTCACCA	1.10E-02	107 (1.3%)
MHe-30	GCCCATTCC	1.10E-02	101 (1.3%)
MHe-31	ACTGGAGGGTCC	1.50E-02	71 (0.9%)
MHe-32	GCGCGCGCGCGCGC	1.50E-02	86 (1.1%)
MHe-33	CGATTAAACGTTC	1.50E-02	43 (0.5%)
MHe-34	ACCGACCGAC	1.60E-02	768 (9.6%)
MHe-35	AGGCAAAM	1.70E-02	1619 (20.2%)
MHe-36	TGYGCANTGCRCA	1.70E-02	179 (2.2%)
MHe-37	ACCTTGCGCTA	1.90E-02	112 (1.4%)
MHe-38	CTCCTCCTWC	2.30E-02	673 (8.4%)
MHe-39	CAACCAATCAA	2.90E-02	465 (5.8%)
MHe-40	ACCGAATTAAGCACT	3.10E-02	85 (1.1%)
MHe-41	ACCACAAGACTGC	3.10E-02	66 (0.8%)
MHe-42	AATCGGMTCACTYRT	3.10E-02	75 (0.9%)
MHe-43	AACGAGTGTA	3.10E-02	61 (0.8%)
MHe-44	GAGAAGAAA	3.30E-02	1080 (13.5%)
MHe-45	VGTACKMGTACB	3.50E-02	93 (1.2%)
MHe-46	ATTCCAMTTCCA	3.80E-02	150 (1.9%)
MHe-47	AGSCARGCAK	3.80E-02	157 (2.0%)
MHe-48	ACCACGCCC	3.80E-02	135 (1.7%)
MHe-49	AAACAAACA	4.60E-02	1353 (16.9%)

Table S2 Motif similarity to TF binding site database

Query motif	p-value	Target_consensus	class	TF name	species
MRv-6	6.00E-04	AAAAAGAGGAAGTGGAA	Tryptophan cluster factors	Spi1	*Mus musculus*
	6.00E-04	AAAAAGAGGAAGTA	Tryptophan cluster factors	*SPIC*	*Homo sapiens*
	8.00E-04	AAAAGAGGAAGTGAAA	Tryptophan cluster factors	SPIB	*Homo sapiens*
	2.00E-03	AAGAGAAAGAA	C2H2 zinc finger factors	PRDM1	*Homo sapiens*
	3.00E-03	GGAGGAGGTGAGAA	C2H2 zinc finger factors	ZKSCAN5	*Homo sapiens*
MRv-7	5.00E-04	GGTGGGCAGGGAGGAAGCAGAAGG	C2H2 zinc finger factors	PRDM9	*Homo sapiens*
	7.00E-04	AGGCGGAAGTG	Tryptophan cluster factors	ETV7	*Homo sapiens*
	9.00E-04	GGCGGAAGCAGGTGG	Tryptophan cluster factors ::Basic helix-loop-helix factors	ETV5::FIGLA	*Homo sapiens*
	2.00E-03	GGAGGAGGTGAGAA	C2H2 zinc finger factors	ZKSCAN5	*Homo sapiens*
	2.00E-03	AGAAGGAAGTGA	Tryptophan cluster factors	Elf5	*Mus musculus*
MRv-39	1.00E-03	CGGAAAAAT	C6 zinc cluster factors	EDS1	*Saccharomyces cerevisiae*
	1.00E-03	CACAAAACGACAAAA	C2H2 zinc finger factors	GAF1	*Arabidopsis thaliana*
	2.00E-03	CACAAAACGACAAAAA	C2H2 zinc finger factors	IDD5	*Arabidopsis thaliana*
	2.00E-03	CACAAAACGACAAAAAAA	C2H2 zinc finger factors	IDD7	*Arabidopsis thaliana*
	3.00E-03	CCGGAAAAATT	C6 zinc cluster factors	RGT1	*Saccharomyces cerevisiae*
MHe-2	3.00E-09	GAGAGAGAGAGAGA	C2H2 zinc finger factors	RAMOSA1	Zea mays
	3.00E-08	GAGAGAGAGAGAGAGAGAGAGAGA	BBR/BPC	BPC1	*Arabidopsis thaliana*
	6.00E-08	GAGAGAGAGAGAGAGAGAG	BBR/BPC	BPC6	*Arabidopsis thaliana*
	2.00E-07	AGAGAGAGAGAGAGAGAGAGAGAGAGAG	BBR/BPC	BPC5	*Arabidopsis thaliana*
	1.00E-06	AGAGAGACGCAGAGA	C2H2 zinc finger factors	eor-1	Caenorhabditis elegans
MHe-9	5.00E-04	GTCCAAGAAGAATGA	C2H2 zinc finger factors	ZNF680	*Homo sapiens*
	1.00E-03	GGAAGGAAGGAAGGAAGG	Tryptophan cluster factors	EWSR1-FLI1	*Homo sapiens*
	2.00E-03	AAAAAGAGGAAGTA	Tryptophan cluster factors	SPIC	*Homo sapiens*
	2.00E-03	ATGAAGAAACCTA	GCM domain factors	NTL8	*Arabidopsis thaliana*
	3.00E-03	GTAAACAGGAAGTA	Fork head/winged helix factors ::Tryptophan cluster factors	FOXO1::ELF1	*Homo sapiens*
MHe-35	3.00E-04	AAAAAAAAAAGGCAAAAAA	Other C4 zinc finger-type factors	DOF4.2	*Arabidopsis thaliana*
	1.00E-03	AAAGTCAAAA	GCM domain factors	WRKY40	*Arabidopsis thaliana*
	2.00E-03	AAAAAAAGACAAAAA	C2H2 zinc finger factors	SGR5	*Arabidopsis thaliana*
	2.00E-03	AAAAAAAGCAAAAAGTAAA	Other C4 zinc finger-type factors	DOF3.2	*Arabidopsis thaliana*
	2.00E-03	AAAGTTAGGCAGA	Tryptophan cluster factors	MYB116	*Arabidopsis thaliana*
